# Case report: rapid spontaneous recovery from severe hypothyroidism in 2 teenage girls

**DOI:** 10.1186/1687-9856-2012-9

**Published:** 2012-05-02

**Authors:** Paul B Kaplowitz

**Affiliations:** 1 Division of Endocrinology, Children’s National Medical Center, 111 Michigan Ave NW, Washington, 20010, DC, USA; 2 Pediatrics, George Washington University School of Medicine and the Health Sciences, Washington, DC, USA

## Abstract

**Background:**

While it is recognized that patients sometimes recover from autoimmune hypothyroidism, little is known about how rapidly this may occur.

**Case reports:**

Two 13 year old girls had severe primary hypothyroidism (total T4 14.2 nmol/L with TSH 468 miU/L and total T4 7.7 nmol/L with TSH 183 miU/L) accompanied by goiter and positive thyroid peroxidase antibodies. There were delays in starting thyroid hormone replacement, and complete reversal of hypothyroidism was documented within 2 months in both cases. One of the girls had recurrence of severe hypothyroidism after being euthyroid for 18 months.

**Review of literature:**

There are few published studies which have looked systematically at reversibility of acquired hypothyroidism, but one Japanese study found that recovery from autoimmune hypothyroidism may occur within weeks. Other causes of primary hypothyroidism (TSH-blocking antibodies, iodine excess, medications) seem less likely, so this probably represents rapid spontaneous reversal of autoimmune hypothyroidism.

**Conclusion:**

Patients with severe autoimmune hypothyroidism may have spontaneous normalization of thyroid tests within weeks to months after diagnosis. This suggests that reevaluating the need for thyroid hormone replacement in selected patients with persistently normal TSH during therapy should be considered.

## Introduction

The natural history of severe hypothyroidism due to autoimmune thyroiditis is generally permanent hypothyroidism and a lifelong need for thyroid hormone replacement. While subclinical and mild hypothyroidism often resolve over time and may not require long-term therapy [1], there is less information on the reversibility of severe primary hypothyroidism. Several older articles mention the that recovery does occasionally occur [2,3], but there is little to guide the clinician on when and how often this may happen. This may be in part because physicians are reluctant to discontinue thyroid hormone replacement in patients who had very low levels of T4 or free T4 and very high levels of TSH at the time they were diagnosed.

Recently, two 13 year old girls were seen by the author whose initial thyroid tests showed severe hypothyroidism with positive thyroid antibodies, and in both cases, after a delay in starting therapy, there was spontaneous normalization of thyroid tests within 2 months. The purpose of this report is to summarize these cases, review the literature on reversal of hypothyroidism, and discuss the implications for management of autoimmune hypothyroidism.

### Patients

Patient 1: A 13 year old girl was seen by her primary care physician because she was feeling cold and tired. She had a total T4 of 14.2 nmol/L and a TSH of 468 mIU/L. Endocrinology was not consulted because the mother refused to have her started on treatment until the tests were repeated, which was done 6 weeks later. At this time, her T4 had increased to 86.4 nmol/L and her TSH had decreased to 17.8 mIU/L(Table 1). The author was then consulted and the mother insisted she had not taken any thyroid hormone. As the thyroid tests had improved considerably, it was decided to hold off on treatment until they were repeated. It is of note that the non-identical twin sister had also been diagnosed with hypothyroidism 7 months earlier when she had a goiter but no obvious symptoms of hypothyroidism, a free T4 of 0.65 pmol/L and a TSH of 915 mIU/L.

**Table 1:** Laboratory findings in 2 girls with rapid reversal of severe hypothyroidism

	**Age**	**Total T4** nmol/L*	**Free T4** pmol/L**	**TSH** **miU/L**	**TPO Ab units/ml #**	**Comments**
Case 1	13.0	14.2		468		
	13.1	86.4		17.8		
	13.3		15.5	0.21	630	
	13.9	73.5		4.3		
	14.8	<6.4		262	1432	
Case 1 DZ twin sister	12.5		0.65	915		
	13.9	105		2.45		75 μg T4
	14.0		5.2	77	528	5 weeks off T4
Case 2	11.0	87.7		3.7		
	13.7	7.7		183		T3 0.85 nmol/L
	13.8		11.6	15.8	291	
	13.9	69.7	13.8	3.55		
	14.5	78.5		5.1		

*Normal range 65–155 nmol/L.

**Normal range 11.6–20.6 pmol/L.

#Normal range <35 units/ml.

When seen 2 months after initial testing, the patient reported that her energy level had improved, and her exam was normal except for a small goiter. There was no history of exposure to high levels of iodide or any goitrogen. She now had a normal free T4 of 15.5 pmol/L, a somewhat low TSH of 0.21 mIU/L, and positive thyroid peroxidase (TPO) antibodies. She was seen back 7 months later at which time her total T4 was 73.5 nmol/L, TSH had increased to 4.3 mIU/L, and the gland was no longer obviously enlarged. At follow-up 9 ½ months later, she had no reported symptoms of hypothyroidism but her gland had enlarged and her low T4 of <6.5 nmol/L with TSH 262 mIU/L indicated that she was again hypothyroid. The patient and mother insisted that she had not taken any of her sister’s l-thyroxine during the 1.5 years she was euthyroid.

Since one sister’s severe hypothyroidism had resolved, the mother requested that her non-identical twin sister be given a trial off thyroid hormone after she had been on treatment for 18 months. After 1 month, the free T4 was low at 5.2 pmol/L (11.6-20.6), TSH 77 mIU/L, and TPO antibodies were 528 units/ml. Compared to her initial tests, this indicated only a partial recovery of thyroid function, so she was restarted on l-thyroxine.

Patient 2: A 13 ½ year old girl was being seen for a buttocks infection when the mother requested thyroid tests because she had noted a goiter which had been present for at least a year. Three years earlier she had had a normal T4 and TSH, but now her total T4 was 7.7 nmol/L, T3 55 ng/dl, and TSH 183 mIU/L. The ordering physician did not see the report for about a month, and when it was noted, it was decided to do confirmatory testing before starting treatment. The TSH had decreased to 15.8 mIU/L, her free T4 was slightly low at 11.6 pmol/L and TPO antibodies were positive (Table 1). It was decided to hold off on treatment until the tests could be repeated. Five weeks later, she denied that she had had any symptoms of hypothyroidism 2 ½ months earlier. She had been on no medications at the time of the initial thyroid tests and there was no history of excessive iodine ingestion or goitrogen exposure. She had a completely normal late-pubertal exam except for a small goiter and her thyroid tests were within the normal range. Six months later she still had a goiter but her T4 was normal and TSH was at the high end of the normal range.

## Discussion

There are several possible reasons that severe primary hypothyroidism might resolve over time. These include recovery from autoimmune thyroiditis, disappearance of TSH-receptor blocking antibodies, recovery from hypothyroidism induced by exposure to large amounts of iodine, and cessation of medications which can cause hypothyroidism. Given that our 2 patients had goiter and positive TPO antibodies and no drug or high iodine exposure history, they most likely had autoimmune hypothyroidism. However, there are very few studies documenting reversal of severe autoimmune hypothyroidism over a time interval as short as that reported here.

One study from Japan is of particular interest in that, due to a high incidence of reversible hypothyroidism from excess iodine ingestion, their protocol involved asking patients to refrain from iodine-containing medication or high iodine foods like seaweed for 2–15 weeks while thyroid tests were monitored before starting thyroid hormone. Between 1977 and 1991, 249 patients with primary hypothyroidism (TSH >40 mIU/L) were seen in one department, of whom 91 non post-partum patients had ≥ 50% decrease in TSH during iodine restriction [4]. These were categorized, based presence of thyroid antibodies or either elevated serum non-hormonal iodine or history of excess iodine ingestion, as being chemical (n = 28), immunological (n = 20) or mixed (n = 43) hypothyroidism, Chemical hypothyroidism recovered rapidly with iodine restriction (mean 6.1 days) whereas recovery was slower (mean of 16.8 days and 12.7 days for a 50% decrease in TSH) for the immunological and mixed hypothyroidism groups. In the immunological group there were patients in whom TSH decreased from >100 mU/L to <10 over a period of 2–3 months, presumably reflecting the natural history of their autoimmune thyroiditis, as there is no reason to believe iodine restriction was a factor in the thyroid recovery of these subjects. It should be noted that relapse to overt hypothyroidism was noted in 35-38% of subjects with immunological and mixed hypothyroidism but in only 5% of those with chemical hypothyroidism. Patient 1 clearly indicates the potential for recurrence after normalization of thyroid tests.

Another study from Japan looked specifically for cases of acquired hypothyroidism accompanied by TSH receptor blocking antibodies, of which 7 had goiters and 14 were atrophic. Fifteen patients had disappearance of antibodies when followed over a period of 6–10 years. Of these, 6 patients, including 4 with goiter, 5 cases with TSH of <100 mIU/L and one case with TSH of > 300, had normal thyroid tests 1–3 months after stopping l-thyroxine [5]. It took 1–5 years for TSH receptor antibodies to disappear, so given the rapid recovery from hypothyroidism in the 2 cases described above, this mechanism seems less likely. However, it cannot be excluded, since TSH receptor antibodies were not tested at the time the patients were hypothyroid.

Knowing that patients can recover from severe autoimmune hypothyroidism even before the start of replacement therapy when it is delayed may have implications for the long-term management of these patients. While it is not suggested that starting replacement therapy be delayed in order to recheck thyroid tests, it is important to recog-nize that reversal of severe autoimmune hypothyroidism does occur in an unknown proportion of cases. One important question is whether there is any way to predict which hypothyroid patients have had recovery of thyroid function and can be withdrawn from therapy. One study of 92 patients with Hashimoto’s thyroiditis on thyroid replacement looked at the TSH and l-thyroxine responses to administration of 500 mcg of thyrotropin releasing hormone (TRH). All 22 patients who had a normal increase in both T4 and TSH after TRH were euthyroid after treatment was stopped and remained euthyroid for 1–8 years, while all 70 patients who did not show thyroid responsiveness to a TRH-induced rise in TSH became hypothyroid within 3 months of being taken off treatment [6]. However, the lack of commercial availability of TRH would make this test difficult to perform. One possible clinical indicator of recovery is having a TSH consistently in the mid or low-normal range on a smaller dose than is typical for the age and size of the patient, which may suggest at least some residual thyroid function.

It is difficult to estimate the frequency with which spontaneous reversal of severe autoimmune hypothyroidism occurs, although the author has seen at least 3 other cases in which severely hypothyroid patients discontinued treatment on their own and were found to be either euthyroid or only mildly hypothyroid. Studies are needed in which large groups of children and adults with autoimmune hypothyroidism and persistently normal TSH on treatment are systematically taken off for 1–2 months and retested. This would reveal the frequency of reversal of hypothyroidism, and would help identify factors (e.g. age, sex, presence of goiter, thyroid antibody levels, and dose of l-thyroxine) which predict a higher likelihood of reversal. At present, this option may be offered to patients with persistently normal TSH during replacement therapy who wish to find out if their hypothyroidism is permanent. It has been noted that hypothyroidism may redevelop in patients who have recovered from autoimmune hypothyroidism [4], which was clearly demonstrated in patient 1 even in the absence of symptoms of hypothyroidism, so periodic follow-up testing of such patients is necessary.

## Competing interests

The author declares that he has no competing interests.
